# Epidemiological characteristics of leprosy from 2000 to 2019 in a state with low endemicity in southern Brazil^☆^

**DOI:** 10.1016/j.abd.2022.08.009

**Published:** 2023-04-27

**Authors:** Paulo Cezar de Moraes, Letícia Maria Eidt, Alessandra Koehler, Leonardo Girardi Ransan, Maria Lúcia Scrofeneker

**Affiliations:** a Department of Sanitary Dermatology, Sanitary Dermatology Outpatient Clinic, State Health Secretariat of Rio Grande do Sul, Porto Alegre, RS, Brazil; b Department of Microbiology, Immunology and Parasitology, Universidade Federal do Rio Grande do Sul, Porto Alegre, RS, Brazil; c Postgraduation Program in Medicine ‒ Medical Sciences, Universidade Federal do Rio Grande do Sul, Porto Alegre, RS, Brazil

**Keywords:** Epidemiology, Health profile, Leprosy, Prevalence

## Abstract

**Background:**

Leprosy is an infectious and contagious disease caused by *Mycobacterium leprae* and is mainly characterized by lesions in the skin and peripheral nerves. In Brazil, it is a public health problem due to its high endemicity. However, the state of Rio Grande do Sul presents low endemicity of this disease.

**Objective:**

To characterize the epidemiological profile of leprosy in the state of Rio Grande do Sul from 2000 to 2019.

**Methods:**

This was a retrospective observational study. Epidemiological data were collected from the Notifiable Diseases Information System (SINAN, *Sistema de Informação de Agravos de Notificação*).

**Results:**

Among the 497 municipalities in the state, 357 (71.8%) registered cases of leprosy in the assessed period, with an average of 212 (81.5%) new cases per year. The average detection rate was 1.61 new cases per 100,000 inhabitants. The male sex was predominant (51.9%) and the mean age was 50.4 years. Regarding the epidemiological clinical profile; 79.0% of the patients were multibacillary; 37.5% presented the borderline clinical form; 16% had grade 2 physical disability at diagnosis and bacilloscopy was positive in 35.4% of cases. As for treatment, 73.8% of the cases were treated with the standard multibacillary therapeutic regimen.

**Study limitations:**

There were missing/inconsistent data in the database available.

**Conclusions:**

The findings observed in this study indicate that the state presents a low endemicity profile of the disease and these results can support adequate health policies relevant to the reality of Rio Grande do Sul, inserted in a national scenario of highly endemic leprosy.

## Introduction

Leprosy is an infectious disease caused by *Mycobacterium leprae*, which is characterized mainly by lesions in the skin and peripheral nerves. As the disease progresses, it can lead to damage to other organs such as the liver, kidneys, lymph nodes, testes, eyes, and lymph nodes.^1^, ^2^

The first records of leprosy in history were made in 600 BC in China and its first clinical description was made in the 3^rd^ century BC.^3^, ^4^ Leprosy is a disease with worldwide distribution that still causes prejudice and psychological suffering, and the patient is viewed with disapproval by the population.^1^, ^2^, ^3^, ^4^ Since the introduction of multidrug therapy with the possibility of a cure in 1982, there has been a significant reduction in the number of cases.^4^ The World Health Organization (WHO), in 1991, at the 44^th^ session of the World Health Assembly, approved a resolution that established as a goal the elimination of leprosy as a public health problem by the year 2000. For the goal to be accomplished, it was necessary for the disease prevalence to be less than one case per 10,000 inhabitants.^5^, ^6^

India is the country with the highest number of leprosy cases, with Brazil ranking second, due to high endemicity.^4^, ^7^, ^8^ There were 301,638 new cases in the country from 2010 to 2019.^9^ The detection rate decreased by 37% over time and, in 2019, it was 13.23 cases per 100,000 inhabitants. In the Northern, Northeastern, and Midwestern regions, the disease is of high endemicity, while the Southern and Southeastern regions present a medium level of endemicity, with Rio Grande do Sul and Santa Catarina being classified as states with low endemicity.^9^ For the classification of endemicity, one must consider the following rates per 100,000 inhabitants: > 40.00 cases (hyperendemicity); 20.00 to 39.99 cases (very high endemicity); 10.00 to 19.99 cases (high endemicity); 2.00 to 9.99 cases (medium endemicity) and < 2.00 cases (low endemicity).^2^

According to the last census in 2010, the state of Rio Grande do Sul has a population of 10,693,929 people, distributed into 497 municipalities, with around 15% of individuals residing in rural areas, and with a human development index of 0.746.^10^ Rio Grande do Sul, during the period of 2010 to 2019, remained a state with low endemicity, recording an average of 1.16 cases per 100,000 inhabitants.^2^ The classification of low endemicity should not be considered a comfortable situation meaning resolution of the disease; other parameters must be observed such as the occurrence of cases in children under 15 years of age,^11^, ^12^ clinical form of the disease, percentage of physical disability degree at diagnosis and percentage of cured cases.^2^ It is also necessary to evaluate the health care network for people with leprosy, its coverage, and capacity for diagnosis and treatment, evaluation of contacts, and follow-up after treatment when necessary. Currently, services are offered in response to demand and, therefore, diseases that are apparently under control and with lower occurrence rates are ultimately neglected in government policies.

The study aimed to characterize the epidemiological profile of leprosy in the state of Rio Grande do Sul and correlate elements that contribute to bringing public policies closer to the reality of the present situation in a state with low endemicity of leprosy.

## Methods

This was a retrospective observational study, carried out in the State of Rio Grande do Sul (32°1′60″ South, 52°5′55″ West), located in southern Brazil, from 2000 to 2019.

The epidemiological data were collected from the Notifiable Diseases Information System (SINAN, *Sistema de Informação de Agravos de Notificação*) in July 2020, which was made available by the State Health Surveillance Center of Rio Grande do Sul. Information on the state population was obtained through the 2010 Demographic Census, the last one carried out to date by the Brazilian Institute of Geography and Statistics (IBGE, *Instituto Brasileiro de Geografia e Estatística*). The detection and prevalence rates presented herein followed the pattern recommended by the Ministry of Health, which are the detection coefficient per 100,000 inhabitants and the prevalence coefficient per 10,000 inhabitants.^2^

Data processing was carried out through a database in an Excel 365 spreadsheet with sociodemographic indicators (sex, age group, race/skin color, level of schooling, area of residence) and clinical epidemiological indicators (operational classification, clinical classification, entry mode, detection mode, degree of physical disability, bacilloscopy and therapy). To calculate the detection and prevalence rates, according to Ministry of Health guidelines,^2^ only cases classified as “new cases” in the case entry mode were considered. The other sociodemographic and clinical indicators were analyzed in the total number of cases, regardless of the entry mode.

Statistical analyses were performed using the SPSS 26 software. The descriptive statistical measures used were rates, frequency, percentage, mean and standard deviation. The bivariate analyses were performed using the Chi-square test for categorical variables, using a statistical significance level of 5% (two-tailed).

The research project was submitted to the Research Ethics Committee of Hospital de Clínicas de Porto Alegre (CAAE: 31959120.6.0000.5327) and to the Ethics Committee of Escola de Saúde Pública do Rio Grande do Sul (CAAE: 3195120.6.5312), being approved in accordance with Counsels n. 4.075.445 and 4.121.621, respectively, according to Resolutions 466/12 and 510/16 of the National Health Council that regulate research with human beings.

## Results

From 2000 to 2019, Rio Grande do Sul presented an average of 212 cases per year, with 81.6% (n = 3,442) being new cases. The average rate of detection of new cases was 1.61 cases per 100,000 inhabitants/year. Among children under 15 years of age, this rate was 0.04 cases per 100,000 inhabitants/year, corresponding, on average, to 2.48% of the total number of new cases in the studied period. The leprosy prevalence rate in the study was always less than one case per 10,000 inhabitants, with an average of 0.16 cases per 10,000 inhabitants (Table 1). Over the period, the average number of new cases classified as multibacillary was 131.2 cases. Thus, on average, 76.2% of the diagnoses performed per year, during the studied period, were multibacillary ones. The rate of visible physical disability degree (Grade 2), already present at the diagnosis, ranged between 0.09 and 0.50 cases per 100,000 inhabitants. On average, the rate was 0.22 cases per 100,000 inhabitants/year (Table 1).

**Table 1:** Leprosy control indicators in Rio Grande do Sul, according to the Brazilian Ministry of Health, distributed by year, from 2000 to 2019

Reference year	Detection rate (per 100,000 inhabitants)	Detection rate in children under 15 years of age (per 100,000 inhabitants)	Cases in children under 15 years of age (%)	Multibacillary cases	Rate of new cases with grade 2 at diagnosis (per 100,000 inhabitants)	Prevalence rate (per 10,000 inhabitants)
2000	1.80	0.04	2.07	136	0.50	0.18
2001	1.88	0.02	1.00	125	0.16	0.19
2002	2.30	0.07	2.85	176	0.31	0.23
2003	2.20	0.05	2.13	173	0.14	0.22
2004	2.38	0.07	2.76	197	0.22	0.24
2005	2.35	0.04	1.59	202	0.36	0.23
2006	1.89	0.01	0.50	150	0.20	0.19
2007	1.78	0.04	2.11	152	0.28	0.18
2008	1.80	0.01	0.52	135	0.26	0.18
2009	1.57	0.04	2.38	126	0.13	0.16
2010	1.34	0.04	2.80	109	0.09	0.13
2011	1.37	0.03	2.05	113	0.20	0.14
2012	1.45	0.01	0.65	121	0.20	0.14
2013	1.45	0.02	1.29	111	0.18	0.14
2014	1.41	0.03	1.99	116	0.22	0.14
2015	1.10	0.03	2.54	101	0.18	0.11
2016	0.98	0.02	1.90	94	0.17	0.10
2017	1.05	0.07	6.25	98	0.20	0.10
2018	1.10	0.05	4.24	106	0.27	0.11
2019	0.99	0.07	7.55	83	0.20	0.10

**Figure 1:**
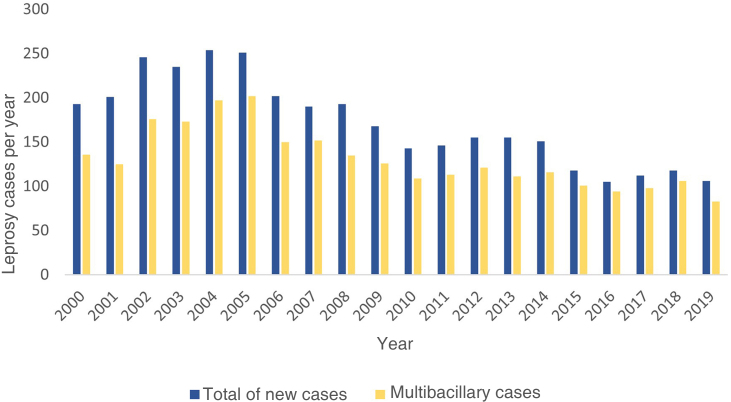
Numbers of new cases of leprosy notified from 2000 to 2019, and multibacillary cases in the same period, according to year, in the state of Rio Grande do Sul, Brazil

The period with the highest number of records in this section occurred from 2000 to 2006, with an average of 226 new cases per year. As of 2007, a downward trend occurred in the number of new cases, to below 200 new cases/year, with an average of 162.6 new cases/year until 2014, representing a reduction of 38.4%. From 2015 to 2019, this decline increased to 50.5%, compared to the period between 2000 and 2006. From 2006 onwards, there was some stability in the registration of new cases, which remained at an average of 112 cases per year until 2019. There was a predominance of multibacillary cases, a clinical form present in at least 69% of cases/year (Fig. 1).

**Figure 2:**
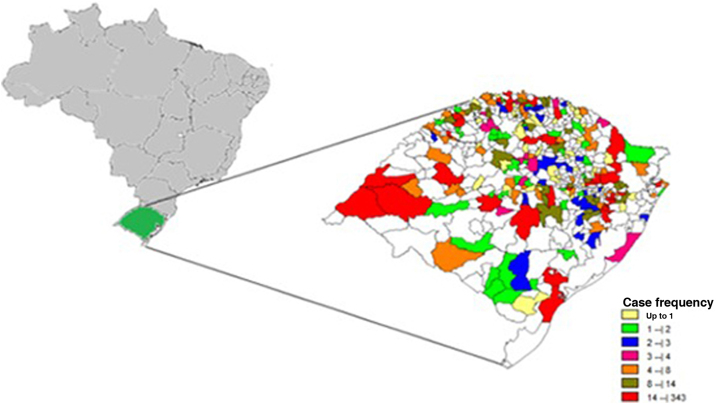
Georeferencing of notified leprosy cases from 2000 to 2019, according to the place of residence, in the state of Rio Grande do Sul, Brazil. Source: the authors

Among a total of 497 municipalities, 357 (71.9%) recorded cases of leprosy. The municipalities with the highest number of cases among their residents were, in descending order, Porto Alegre (n = 342), São Borja (n = 212), and Uruguaiana (n = 193; Fig. 2).

The male sex was predominant (51.9%) and the overall mean age was 50.4 years (SD = 16.2), and, regarding this variable, there was no difference in relation to sex. When skin color was analyzed, whites were predominant (81.7%). Illiteracy rate was 7.1%, where the noteworthy fact was that 72.7% of patients had not finished Elementary School at the time of the diagnosis. Most patients lived in urban areas (84.3%; Table 2).

**Table 2:** Sociodemographic profile of leprosy from 2000 to 2019, according to sex, in the state of Rio Grande do Sul, Brazil

Characteristics	Sex		Total	%^a^
	Female	Male	n = 4233	
Age group				
0‒14	34 (0.8)	43 (1.1)	77	1.9
15‒29	219 (5.3)	234 (5.6)	453	10.9
30‒49	623 (15.0)	664 (16.0)	1287	31.0
50‒60	579 (14.0)	591 (14.2)	1170	28.2
61‒79	495 (11.9)	581 (14.0)	1076	25.9
80 or older	50 (1.2)	38 (0.9)	88	2.1
Missing data	‒	‒	82	‒
Race/Skin color				
White	1558 (41.0)	1544 (40.7)	3102	81.7
Black	76 (2.0)	110 (2.9)	186	4.9
Brown	184 (4.8)	283 (7.5)	467	12.3
Yellow	11 (0.2)	16 (0.4)	27	0.7
Indigenous	6 (0.2)	9 (0.2)	15	0.4
Missing data	‒	‒	436	‒
Level of schooling				
Illiterate	117 (3.3)	137 (3.8)	254	7.1
1^st^ to 4^th^ grade of Elementary School	530 (14.8)	614 (17.1)	1144	31.9
5^th^ to 8^th^ grade of Elementary School	581 (16.2)	627 (17.5)	1208	33.7
Complete Elementary School	87 (2.4)	107 (3.0)	194	5.4
Incomplete High School	164 (4.6)	181 (5.0)	345	9.6
Complete High School	106 (2.9)	74 (2.1)	180	5.0
Incomplete Higher Education	23 (0.6)	26 (0.8)	49	1.4
Complete Higher Education	133 (3.7)	80 (2.2)	213	5.9
Missing data	‒	‒	646	‒
Area of residence				
Urban	1680 (42.4)	1664 (42.0)	3344	84.4
Rural	265 (6.7)	310 (7.8)	575	14.5
Peri-urban	17 (0.4)	22 (0.6)	39	1.0
Missing data	‒	‒	557	‒

a Percentages were calculated disregarding missing data.

Most leprosy cases in the assessed period were classified as multibacillary (79.0%), with males accounting for 43.4% of these cases. The predominant clinical form was borderline (37.5%), with predominance of females (19.2%). The mode of entry of cases, in almost all of them (81.6%), comprised new cases. Recurrence cases represented 7.8% of the sample, and the method of detection was referral in 52.6% of them. The degree of visible physical disability was already Grade 2 in 16% of the cases at diagnosis, and males were the most affected. Bacilloscopy was performed in 68% of the diagnosed cases, with a positive result in 35.4% of the tests (Table 3).

**Table 3:** Epidemiological clinical profile of leprosy from 2000 to 2019, according to sex, in the state of Rio Grande do Sul, Brazil

Characteristics	Sex		Total	%^a^
	Female n (%)	Male n (%)	n = 4233	
Operational classification				
Paucibacillary	523 (12.4)	359 (8.6)	882	21.0
Multibacillary	1496 (35.6)	1822 (43.4)	3318	79.0
Missing data	‒	‒	33	‒
Clinical form				
Indeterminate	219 (5.9)	169 (4.6)	388	10.5
Tuberculoid	313 (8.5)	231 (6.2)	544	14.7
Borderline	713 (19.2)	680 (18.3)	1393	37.5
Lepromatous	534 (14.4)	851 (22.9)	1385	37.3
Missing data	‒	‒	525	‒
Therapeutic regimen				
Multidrug therapy 6 doses MDT/PB	510 (12.5)	344 (8.2)	854	20.4
Multidrug therapy 12 doses MDT/MB	1393 (33.2)	1703 (40.6)	3096	73.8
Substitute regimens	116 (2.8)	130 (3.0)	246	5.8
Missing data	‒	‒	37	‒
Case entry mode				
New case	1690 (40.1)	1752 (41.5)	3442	81.6
Recurrence	149 (3.5)	179 (4.3)	328	7.8
Other modes	192 (4.6)	255 (6.0)	447	10.6
Missing data	‒	‒	16	‒
Case detection mode				
Referral	870 (24,8)	976 (27,8)	1846	52,6
Spontaneous demand	597 (17,0)	614 (17,5)	1211	34,5
Collective examination	12 (0,4)	22 (0,6)	34	1,0
Examination of contacts	216 (6,1)	136 (3,9)	352	10,0
Other modes	30 (0,9)	35 (1,0)	65	1,9
Missing data	–	–		–
Assessment of the degree of physical disability				
Grade zero	1016 (26,3)	911 (23,5)	1927	49,8
Grade 1	622 (16,0)	700 (18,2)	1322	34,2
Grade 2	252 (6,4)	370 (9,6)	622	16,0
Missing data	‒	‒	362	‒
Bacilloscopy				
Negative	379 (18,1)	302 (14,5)	681	32,6
Positive	261 (12,5)	477 (22,9)	738	35,4
Not performed	311 (14,9)	358 (17,1)	669	32,0
Missing data	‒	‒	2145	‒

a Percentages were calculated disregarding missing data.

In the bivariate analysis, an association (p ≤ 0.001) was found between multibacillary classification and physical disability Grade 2, with the same fact being verified for the borderline and lepromatous clinical forms (p ≤ 0.001). Regarding bacilloscopy, a significant association (p ≤ 0.001) was also found with physical disability Grade 2 (Table 4).

**Table 4:** Cross-quantitative distribution, associating the characteristics: operational classification, clinical forms and bacilloscopy with degree of physical disability of leprosy, from 2000 to 2019, in the state of Rio Grande do Sul, Brazil

Characteristics^a^	Degree of physical disability		p -value^d^
	Grade 0 or 1 (n = 3237)	Grade 2 (n = 622)	
	n (%)	n (%)	
Operational classification			<0.001
Paucibacillary	779 (24.0)	33 (5.3)	
Multibacillary	2458 (76.0)	589 (94.7)	
Clinical form^b^			<0.001
Indeterminate	348 (12.0)	11 (1.9)	
Tuberculoid	467 (16.1)	43 (7.4)	
Borderline	1097 (38.0)	221 (38.2)	
Lepromatous	979 (33.9)	303 (52.4)	
Bacilloscopy^c^			<0.001
Negative	528 (51.7)	103 (38.6)	
Positive	494 (48.3)	164 (61.4)	

a Total data may vary depending on the possibility of missing data and non-response.

b Clinical form: no visible physical disabilities n = 2891; with visible physical disabilities n = 578.

c Bacilloscopy: no visible physical disabilities n = 1022; with visible physical disabilities n = 267.

d Chi-Square Test.

## Discussion

Rio Grande do Sul is a state with low endemicity for leprosy. However, in the assessed period, there was at least one recorded case in more than 70% of its municipalities. With regard to sex, other studies also reported the predominance of males, with a small difference in percentage.^13^, ^14^ A study was carried out in Rwanda, Central Africa, with a smaller number of cases (77 patients), which showed that 75% of the patients were male.^15^ However, other authors found a predominance of females in their analysis,^16^, ^17^, ^18^ indicating that there is sex variation according to time and place. Regarding age groups, there was a prevalence of cases between 30 and 60 years (59.2% of cases), which constitute the economically active age groups. The predominance of cases in these age groups, between 30 and 60 years old, as well as in persons white skin color, is in agreement with data found in the literature.^16^, ^19^, ^20^, ^21^

The predominance of the white skin color in the present study should not be considered relevant because these data reflect only the predominant skin color in the population of the southern region of Brazil. In Rio Grande do Sul, according to the Brazilian Institute of Geography and Statistics (IBGE, *Instituto Brasileiro de Geografia e Estatística*), 85.9% of respondents self-declared to be white in 2013. Therefore, this skin color is expected to predominate among leprosy cases in the state.^22^ In other studies, the predominant skin color was brown, while the area of residence and economically active age group were similar to those found in the present data.^23^, ^24^, ^25^, ^26^

The next age group with the highest number of cases was between 61 and 79 years (25.9%), which may indicate long disease duration and late diagnosis. In this age group, leprosy may be associated with comorbidities, and it is necessary to observe the occurrence of other diseases and drug interactions.^26^, ^27^

As for the level of schooling, not having finished Elementary School was similar to that found in other studies.^16^, ^19^, ^20^, ^21^, ^23^ As for the area of residence, 84.3% of patients lived in urban areas, data that corroborate the population characteristics of the state of Rio Grande do Sul, where only 15% of people live in rural areas.^10^ The sociodemographic variables are similar in different regions of Brazil, with a predominance of males, economically active age group, low level of schooling, and urban residence, with only a certain variation in skin color depending on the region.^16^, ^19^, ^20^, ^21^, ^23^

There was a predominance of cases classified as multibacillary, and only 21% of cases were paucibacillary, corroborating the literature data.^16^, ^19^, ^21^ However, in endemic regions, some studies show that most cases are classified as paucibacillary.^20^, ^28^ The operational classification showed to be consistent data in SINAN, as it was absent in only 0.7% (n = 33) of the records. However, in the present study, indeterminate and tuberculoid clinical forms, which are included in the paucibacillary classification, represented 25.2% of the cases when combined, while the total number of cases classified as paucibacillary was only 21.0%. This demonstrates inconsistency in data recording. This occurrence can be explained in part by substitutive treatments, which were carried out in 5.8% of cases in the total sample. However, it was observed that treatment changes can occur in all clinical forms of the disease.^28^, ^29^ Other possibilities are the inadequate transposition of information to SINAN and errors in filling out the data by the professionals who performed the diagnosis. SINAN also has limitations regarding the standardization of records, duplication of cases, and lack of information. Nevertheless, it is an important source of information for notifiable diseases in Brazil. Data inconsistencies are difficult to verify, considering the model of the present study.

As for the therapeutic regimen, the standard multibacillary regimen was carried out in 73.8% of the cases, and for 5.8% of the leprosy cases, the substitutive therapeutic regimen was indicated at the beginning of treatment, which may indicate previous comorbidities, low weight and previous intolerance to one of the multidrug therapy medications. However, it was not possible to define the reason for choosing the substitutive treatment, because SINAN does not collect this type of information. In other studies, the main drug that results in changes in the standard treatment is dapsone, which has side effects such as hemolytic anemia, liver alterations, and gastrointestinal problems, usually manifesting in the first four months of treatment.^29^, ^30^, ^31^

Regarding the case entry mode, there was a low number of cases diagnosed by evaluation of contacts (10.0%), however there are studies indicating that, for one index case, there is a chance of up to a 2.8-fold possibility of diagnosing new cases among contacts.^32^, ^33^ The lack of evaluation of contacts is one of the factors that may explain the high number of cases with a degree of physical disability already established at the diagnosis (50.2%), an important indicator of late diagnosis and risks for disabilities and sequelae, both physical and emotional.^3^, ^19^, ^20^, ^25^

Bacilloscopy was performed in most cases (68%) which had this information reported, and its main purpose is to determine leprosy classification. When positive, it indicates a multibacillary case, but if negative, it does not allow the diagnosis to be discarded, and the test is not mandatory for disease diagnosis.^2^ Moreover, some multibacillary patients do not have a positive bacilloscopy, even when naïve for treatment. Cavalcanti et al. showed that agreement between bacilloscopy and the operational classification occurred in only 30% of the cases; however, even so, the test positively contributes to the identification of borderline cases. In these cases, the use of the clinical classification alone, according to the number of lesions, would lead multibacillary cases to being misclassified and treated as paucibacillary ones.^2^, ^34^ Bacilloscopy also helps monitoring at the end of treatment, allowing the comparison of the bacillary index at the beginning of treatment and the end.^2^ It is important to emphasize that, in the state of Rio Grande do Sul, this test is only performed at the state reference center for leprosy, the Sanitary Dermatology Outpatient Clinic, located in Porto Alegre.

Over the 20 year period of assessment, the epidemiological behavior of leprosy in the state of Rio Grande do Sul showed low endemicity, with the exception of the period between 2002 and 2005. From 2006 onwards, the annual detection rate was always lower than two cases per 100,000 inhabitants,^2^, ^35^ and the prevalence rate in this period was always lower than one case per 10,000 inhabitants. Thus, the magnitude of leprosy was low, and these data suggest disease stability behavior during the studied period, associated with the disease behavior in children under 15 years of age, with an average of 0.04 cases per 100,000 inhabitants.^2^ However, it is necessary to assess the rate of physical disability degree and the number of multibacillary cases, which may indirectly indicate disease transmission and early diagnosis.^2^, ^36^, ^37^

The association (p < 0.001) of the multibacillary operational classification with the physical disability Grade 1 or 2 suggests that multibacillary patients are more likely to develop a certain degree of physical disability and that early diagnoses, in the paucibacillary forms, constitute a form of protection against the onset of these disabilities.^38^, ^39^, ^40^ In the present study, 94.7% of cases with Grade 2 physical disability at the diagnosis were multibacillary cases, suggesting late diagnosis, the possibility of continuity of disease transmission, and the possibility of reactive outbreaks or sequelae.^24^, ^38^ It is also emphasized that Rio Grande do Sul, according to data from the Ministry of Health, is the state that has the highest rate of physical disability Grades 1 and 2, confirming late diagnosis in the state.^8^, ^9^

The association (p < 0.001) between positive and Grade 2 physical disability highlights that the combination of these two factors at the diagnosis can be decisive for treatment with episodes of reactional outbreaks and physical disability or sequelae.^41^, ^42^ Physical disabilities and sequelae lead to prejudice and social distancing, which increases the possibility of treatment abandonment and/or the need for post-discharge follow-up.^3^

## Conclusion

According to the clinical-epidemiological profile of leprosy in the period of 2000 to 2019, Rio Grande do Sul is a state with low endemicity. However, in the presence of other health demands such as tuberculosis, dengue, hepatitis, and HIV/AIDS, leprosy goes unnoticed by public policies. There is no appropriate care network, as well as regionalized centers for investigation, diagnosis, and treatment of the disease, with only one referral center in the state of Rio Grande do Sul.

In this context, a high number of multibacillary cases with a degree of physical disability already present at diagnosis, positive bacilloscopy, and a low number of leprosy diagnoses in the assessed contacts were observed. Additionally, the high number of diagnoses with a visible degree of disability (Grade 2) and multibacillary cases may suggest the existence of unknown cases in the state, which, in a way, are expected data for locations with low endemicity or undergoing disease elimination. A positive point observed in the present study was the reduced number of leprosy cases in children under 15 years of age, which is an important indicator to measure the level of disease endemicity listed by the Brazilian Ministry of Health.

It is necessary to broaden the discussion of the number of cases identified per year, the existence of unknown cases, the lack of knowledge about the disease by health professionals, and the lack of information in the community about the disease, aiming to strengthen the self-esteem of leprosy patients and raise awareness of public health managers regarding actions aimed at seeking visibility of the disease.

Carrying out epidemiological profiles in regions with low endemicity allows expanding discussions on late diagnosis, physical disabilities, and self-care, especially after diagnosis and treatment, making it possible to know the behavior of the disease and its problems.

Regarding diseases that have heterogeneous prevalence between regions, such as leprosy, it is known that locations with higher incidence and prevalence rates are the focus of actions and attention from the national leprosy control program.

It is the duty of public health researchers to draw attention to this disease, which is part of the group of neglected diseases and which cannot be disregarded as a public health problem in the state of Rio Grande do Sul. It is hoped that the results of the present study will support adequate health policies and public strategies relevant to the reality of low endemicity in Rio Grande do Sul, inserted in a Brazilian scenario of high endemicity.

## Financial support

None declared.

## Authors' contributions

Paulo Cezar de Moraes: Statistical analysis; drafting and editing of the manuscript; collection, analysis, and interpretation of data; critical review of the literature; approval of the final version of the manuscript.

Letícia Maria Eidt: Design and planning of the study; effective participation in research orientation; critical review of the manuscript; approval of the final version of the manuscript.

Alessandra Koehler: Drafting and editing of the manuscript; critical review of the manuscript; approval of the final version of the manuscript.

Leonardo Girardi Ransan: Critical review of the manuscript; approval of the final version of the manuscript.

Maria Lúcia Scroferneker: Design and planning of the study; effective participation in research orientation; critical review of the manuscript; approval of the final version of the manuscript.

## Conflicts of interest

None declared.
